# Intraoperative near infrared functional imaging of rectal cancer using artificial intelligence methods - now and near future state of the art

**DOI:** 10.1007/s00259-024-06731-9

**Published:** 2024-06-11

**Authors:** Patrick A. Boland, N. P. Hardy, A. Moynihan, P. D. McEntee, C. Loo, H. Fenlon, R. A. Cahill

**Affiliations:** 1 UCD Centre for Precision Surgery, School of Medicine, University College Dublin, 47 Eccles Street, Dublin 7, Dublin, Ireland; 2 Department of Colorectal Surgery, Mater Misericordiae University Hospital, Dublin, Ireland; 3 Department of Radiology, Mater Misericordiae University Hospital, Dublin, Ireland

**Keywords:** Rectal cancer, Fluorescence guided surgery (FGS), Indocyanine green, Intraoperative imaging, Dynamic imaging, Artificial intelligence, Digital surgery, Clinical trials

## Abstract

Colorectal cancer remains a major cause of cancer death and morbidity worldwide. Surgery is a major treatment modality for primary and, increasingly, secondary curative therapy. However, with more patients being diagnosed with early stage and premalignant disease manifesting as large polyps, greater accuracy in diagnostic and therapeutic precision is needed right from the time of first endoscopic encounter. Rapid advancements in the field of artificial intelligence (AI), coupled with widespread availability of near infrared imaging (currently based around indocyanine green (ICG)) can enable colonoscopic tissue classification and prognostic stratification for significant polyps, in a similar manner to contemporary dynamic radiological perfusion imaging but with the advantage of being able to do so directly within interventional procedural time frames. It can provide an explainable method for immediate digital biopsies that could guide or even replace traditional forceps biopsies and provide guidance re margins (both areas where current practice is only approximately 80% accurate prior to definitive excision). Here, we discuss the concept and practice of AI enhanced ICG perfusion analysis for rectal cancer surgery while highlighting recent and essential near-future advancements. These include breakthrough developments in computer vision and time series analysis that allow for real-time quantification and classification of fluorescent perfusion signals of rectal cancer tissue intraoperatively that accurately distinguish between normal, benign, and malignant tissues in situ endoscopically, which are now undergoing international prospective validation (the Horizon Europe CLASSICA study). Next stage advancements may include detailed digital characterisation of small rectal malignancy based on intraoperative assessment of specific intratumoral fluorescent signal pattern. This could include T staging and intratumoral molecular process profiling (e.g. regarding angiogenesis, differentiation, inflammatory component, and tumour to stroma ratio) with the potential to accurately predict the microscopic local response to nonsurgical treatment enabling personalised therapy via decision support tools. Such advancements are also applicable to the next generation fluorophores and imaging agents currently emerging from clinical trials. In addition, by providing an understandable, applicable method for detailed tissue characterisation visually, such technology paves the way for acceptance of other AI methodology during surgery including, potentially, deep learning methods based on whole screen/video detailing.

## Introduction

Colorectal cancer is the third most common cancer across genders and is the second most common cause of cancer deaths [1, 2]. Incidence has almost doubled in patients aged under 55 years since the mid-1990s. The proportion of rectal cancers has also increased from 27 to 31% during the same time-period [1]. Despite this, death rates have been declining at approximately 2% per annum since 2011 with improved awareness, screening, and pre-operative imaging, alongside advancements in surgical and systemic therapies all playing important roles [1–3]. However there still are inadequacies apparent around diagnosis and early stratification of patients regarding their optimal treatment modality. This is particularly evident for early-stage cancers and large pre-cancerous polyps. Clinician assessment, radiological staging, and endoscopic biopsy are limited for staging such lesions, with significant risk of over staging or false negative results [4–7]. Clinicians make treatment decisions, such as whether to perform endoscopic local excision including via Transanal Minimally Invasive Surgery (TAMIS), or radical resection based on these imperfect assessments. There remains a major role to be filled around accurately characterising such colorectal lesions.

Both near infrared (NIR) imaging systems and advanced computing power, including artificial intelligence (AI) methods, are becoming more widespread and accessible to the surgical community through the development of advanced imaging stacks. In this context, dynamic imaging at a molecular level, akin to that already established in radiology [8], is a potential step advancement in intraoperative imaging and surgical management of rectal cancer and significant rectal polyps. Here, we review current and emerging intraoperative NIR dynamic fluorescence imaging strategies that can aid in the surgical management of rectal cancer with a focus on real-time digital analysis of Indocyanine Green (ICG).

### Current state of the art re surgery for significant rectal polyps and tumours

Currently, rectal neoplasia is most often diagnosed on the basis of colonoscopy after either patient reported or screen detected discovery of blood in the stool. Aside from direct visualisation and some specific mucosal surface light-based spectral analysis (which is user dependent) at the time of endoscopic identification, tissue samples are needed to confirm significant lesions (i.e. those > 2 cm) as benign or malignant. Endoscopic biopsies are largely limited to the exposed surface area, and suffer sampling error in approximately 20% of cases, if not more [6, 7, 9, 10] as cancerous transformation often occurs alongside and within areas of preceding benign adenoma. Tissue biopsies are also unreliable for ruling out the presence of residual malignant cells following neoadjuvant therapy [11, 12]. The act of biopsy may also induce fibrosis, potentially frustrating subsequent local excision, particularly endoscopic submucosal dissection [13]. When it comes to treatment, full and partial thickness local excision techniques are available. While patient selection is of course key to ensure oncological outcomes are optimised, it is currently not possible to do so with perfect accuracy (additional radiological assessment is only 50% accurate in this regard). While some cancer presence may not automatically preclude local excision (many T1 cancers are curable using such techniques), the plane of excision (whether submucosal or full thickness) and indeed the provision of an intact specimen is crucial in ensuring sufficient marginal clearance has been achieved. In other cases, when local excision is performed for seemingly benign lesions or indeed even apparent T1 cancers, pathological examination of the excision specimen may only then uncover a more advanced cancer which was not obvious beforehand due to inexact staging. Any such cancers may then require radical surgery with or without preceding neoadjuvant treatment. The adverse effect of an additional surgery is clear for the patient, who must come to terms with their initial surgery being unsuccessful and may have already suffered morbidity despite the minimal access approach. There are increased costs for the healthcare system and the potential increase in complexity of the subsequent operation. “Salvage surgery” following unsuccessful local excision may also be associated with poorer outcomes compared to radical resection in the first instance [14], and is associated with higher rates of non-sphincter-preserving surgery [15]. Marginal involvement after local excision exists in up to 20% of lesions treated with local excision which can lead to regrowth of both benign and malignant lesions. For these reasons, there is much interest in providing better tissue characterisation for significant rectal polyps ideally in situ and without tissue disruption. With the knowledge that aberrant vasculature is an early, hallmark change in the transformation of benign to malignant disease, perfusion based analytical methods central to the now established field of image guided surgery (and more significantly fluorescence guided surgery, FGS) could provide the key to better endoscopic tissue discrimination especially when linked to sophisticated computational analytical methods.

### Indocyanine green (ICG) – the archetypal fluorophore for FGS

Indocyanine green (ICG) is a water-soluble fluorescent dye which binds to plasma proteins, most significantly albumin. Developed by Kodak Research laboratories in 1955, it was approved for human use in 1956 and has been used for retinal angiography since the 1970’s [16, 17]. Since then, it has been adopted for various intraoperative uses across a variety of surgical specialities [18–20]. Across these specialties, ICG angiography is dependent on the compound in plasma absorbing NIR and back-emitting fluorescent light of distinctive wavelength (830 nm). ICG is hepatically metabolised, with a half-life of 3–4 min, depending on liver function and it has an excellent safety profile [19]. All major surgical camera manufacturers now offer the capability for near-infrared light illumination and detection.

Currently, within colorectal cancer surgery, ICG with NIR has several established uses with perfusion assessment being particularly noteworthy. Despite recent advances in technology and technique, anastomotic leakage (AL) remains a significant morbidity following the resection and re-joining (anastomosis) of segments of the large intestine, with tissue malperfusion being a significant contributor to this complication [21, 22]. Given ICG’s affinity for albumin after systemic administration, tissue perfusion is visually reflected by the presence of ICG-related fluorescence within whatever tissue is being observed with NIR [23]. The surgeon then makes a judgement based off observation of the rate of inflow into the tissue area of interest. Such evaluation may reassure the surgeon that the tissues under observation are sufficiently perfused and suitable for selection as the point of anastomosis. Alternatively, it may encourage a change in the point of resection/anastomosis to a segment of bowel which exhibits better perfusion based on the surgeon’s own interpretation of ICG flow [24, 25]. Recent meta-analyses suggest a significant reduction in AL rates when ICG is used for anastomotic assessment in this way [25, 26]. This finding has now too been borne out in a large randomised multicentre clinical trial [27] with other major studies also anticipated to report soon [28]. ICG has also been investigated as a lymph node mapping agent after endoscopic submucosal injection [29] but its clinical value in this remains investigational as the crucial concern in present day colorectal cancer surgery is detection of malignancy rather than demonstration of lymphatic physiology alone. Decisions based on ICG perfusion patterns have to date largely been dictated by qualitative assessment undertaken by the surgeon. However rapid technological advances of the 21st century have presented the possibility of quantifying fluorescent signals from ICG allowing enhancement of intraoperative decision making. This has been shown in neurosurgery and plastic surgery where ICG perfusion quantification, coupled with deep learning methods, have produced intraoperative heatmaps capable of providing intraoperative diagnoses and influencing decision making [30–33].

### ICG for intraoperative cancer imaging – current state of the art

In tandem with the above clinical applications, clinicians realised that ICG given systemically also tends to be trapped in malignant deposits leading to many reports regarding its use as a cancer localisation agent for surgery. The 2009 study by *Ishazawa et al.*, first showed nonselective staining and retention of ICG in hepatocellular carcinomas (HCC) and colorectal liver metastases (CRLM) in patients undergoing routine assessment of liver function via fluorescent imaging [34]. Subsequent to this discovery, antecedent dosing (injection before surgery) has been utilised across several specialties for tumour detection including expanded experiences in CRLM [35], HCC [36] and sarcoma [37], amongst others [38, 39]. It has also been used for margin assessment in breast surgery [40, 41].

While the exact mechanism of ICG uptake is unclear, malignant cells are generally avid consumers of substrates in their vicinity with various nonselective cellular and molecular mechanisms that predispose to the relative retention of ICG (and other substances) in comparison to adjacent normal tissue. Especially in non-hepatic tissue sites (the liver is an active ICG concentrator and its mechanisms for ICG trapping around liver lesions are different such as including the compressed zone around CRLM), the enhanced permeability and retention (EPR) effect [42] is the most commonly attributed pathway, especially when ICG is observed within tumour stroma [43–45]. However, alternative mechanisms likely play key roles as well. A group in Imperial College London has demonstrated the dynamic nature of ICG uptake in patients undergoing breast conserving surgery for breast cancer. ICG administered at 5 min prior to excision demonstrated a significantly superior fluorescence signal within excised specimens when compared to that administered 25 min prior to excision. One potential reason for this finding is higher capillary volume within this tumour and higher intravascular ICG concentration shortly after infusion [46]. Active, relatively nonselective cellular processes, such as clathrin-mediated endocytosis and tight-junction regulation, also play roles in tumour retention of ICG via significant malignant cell internalisation over time after initial dosing [47, 48]. As a side point, the existence of these non-selective uptake mechanisms may also account for a proportion of the uptake of proposed, selective receptor-targeted fluorophores in development.

Whilst cancer sensitivity with such an approach has been impressive, specificity issues are troublesome with antecedent dosing. ICG is prone to trap in non-malignant pathologies (such as inflammation, fibrosis, bleeding [49, 50]), and even non-pathological tissues (such as fat). The resulting false positive rates, along with the workflow impact necessary for compound administration many hours in advance of the planned surgery, limit the widespread adoption of this method. In addition, with all point in time tumour localisation, timing of ICG administration must be so ICG concentration within the region of interest is at its maximum relative to surrounding tissues and significantly different to any sites of non-malignant trapping [51]. However, exact timings of operations and, especially, the exact timing of intraoperative encounter with the site being labelled by ICG can be uncertain.

### Dynamic ICG cancer perfusion patterns and curve analysis

**Fig. 1:**
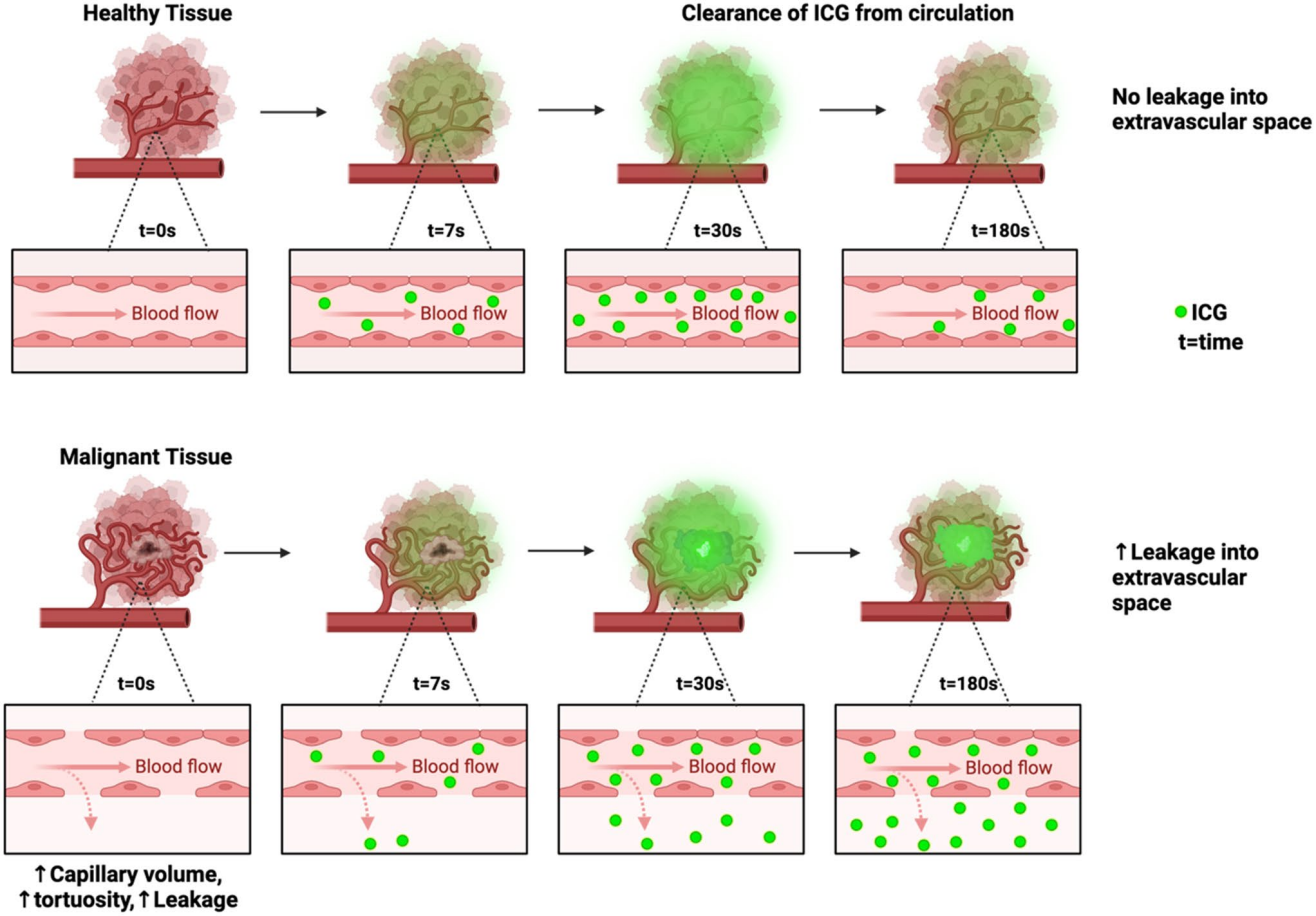
Schematic showing time series changes in ICG after systemic administration in both normal tissue versus a malignant focus in rectal tissue. Among other factors, cancer angiogenesis results in distorted tissue architecture including increased capillary volume and permeability which leads to increased ICG leakage and decreased clearance versus what happens in normal and purely benign lesions. This results in retention of ICG within the malignant core of the polyp when levels have otherwise significantly decreased in normal and benign tissues

**Fig. 2:**
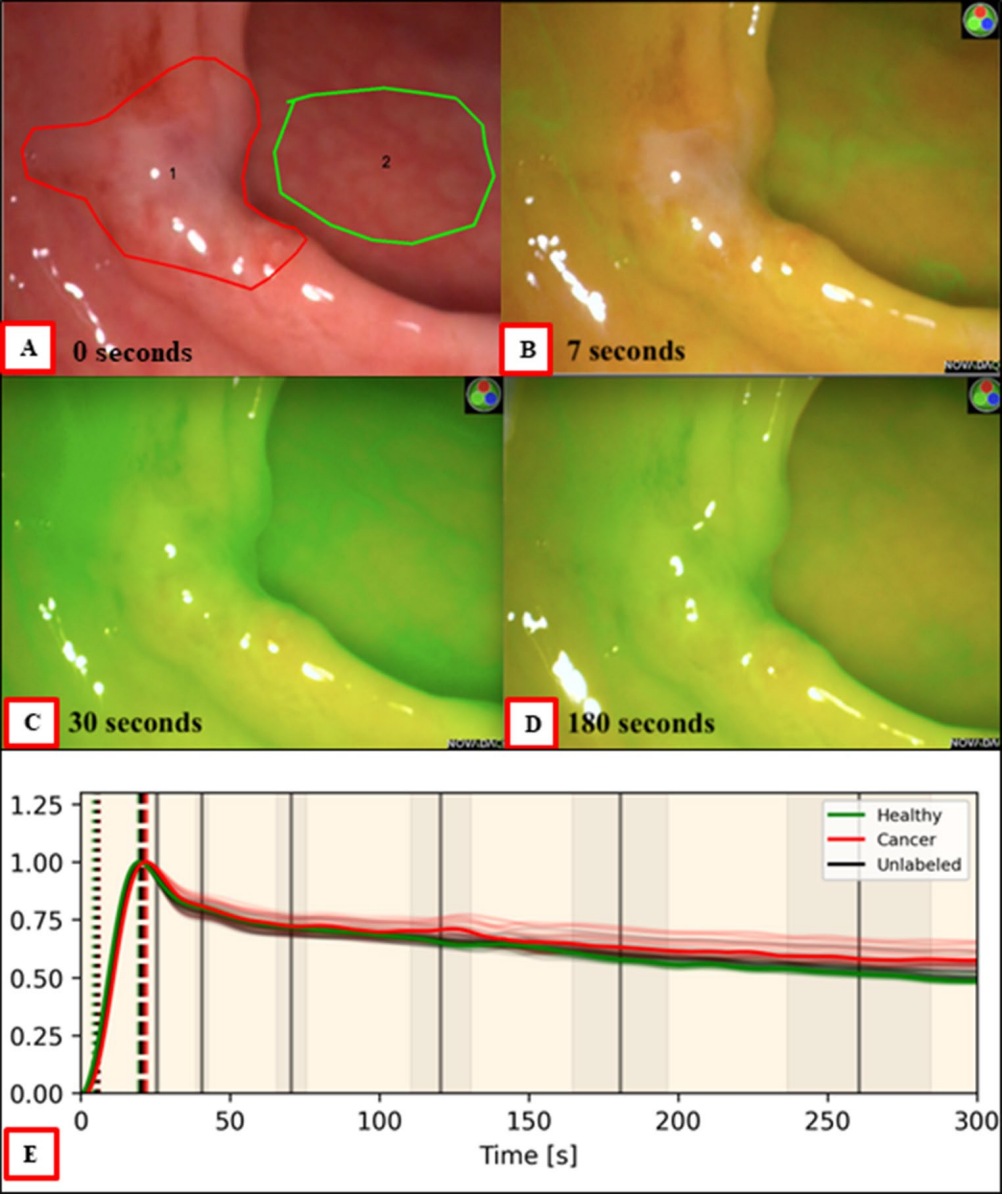
Indicative Progression of ICG fluorescence in a malignant lesion (red border) and health control (green) with a corresponding time series intensity quantification taken from a representative region within the abnormal area compared to a region from the adjacent healthy tissue (interestingly this lesion was initially judged benign by the endoscopist based on clinical impression). Fluorescent appearances are quickly distinctive for malignancy which was confirmed on pathology after its excision. Image **A** shows the subtle area of abnormality seen endoscopically. Image **B** at 7 s from ICG dosage shows nuanced lack of uptake in the tumour relative to the surrounding healthy tissue. At approximately 30 s image **C** shows that the fluorescence of the malignant tissue has now matched or exceeded that of the healthy tissue. Image **D** at 3 min shows significant retention of dye relative to the washout of the healthy tissue. These same trends are more evident in the time series graph (image **E**) demonstrating delayed ICG entry and clearance in the cancerous tissue. Curve features for each case are extracted and serve as the data inputted into machine learning classification pipeline which produces a prediction of “cancer” or “benign”

**Fig. 3:**
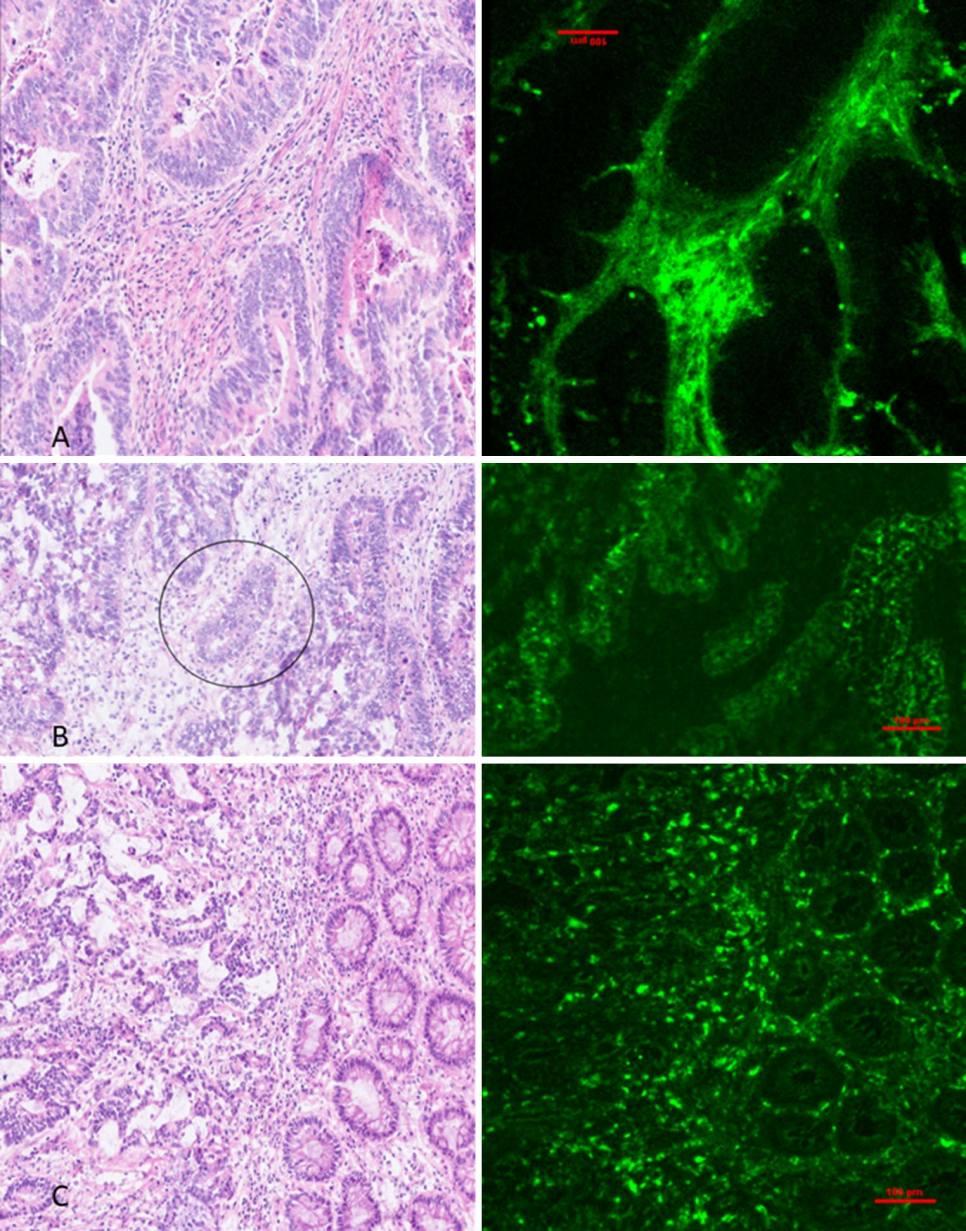
Frozen tissue samples of colorectal cancer (**A**) 15 min and (**B** & **C**) 2 h following systemic indocyanine green injection. Hematoxylin and Eosin stained (left) and microscopic fluorescence imaging of unstained tissue (right) demonstrate a predominance of ICG within stromal tissue and vasculature and a relative lack of ICG within neoplastic malignant glands (circled) at the early timepoint (**A**) Trapping of ICG within the benign: malignant tissue interphase is seen at the later time points **B** & **C**. ICG is also noted to sporadically trap within non-malignant tissues adjacent to cancer, likely as a result of distorted architecture from the nearby malignant process (similar levels of distortion can also be seen in non-malignant inflammatory tissues resulting in false positive appearances associated with single point-in-time ICG assessment). A Nikon Eclipse Ti2 Inverted Research Microscope and a LI-COR Odyssey DLx Near-Infrared *Fluorescence* Imaging System were used to analyse obtained samples. Specimens were mounted in OCT, flash frozen using Lamb’s freezing aerosol and cut using a cryotome to 5 micrometre thick levels

Prior to any uptake into malignant tumours and cells, ICG must be delivered to the lesion via regional blood supply. Distorted and disrupted tissue perfusion is a hallmark characteristic of cancer in general and occurs as an early feature of malignant transformation [52–54]. This provides an important point of differentiation in comparison to surrounding normal tissue. Both malignant and pre-malignant colorectal lesions are also characterised by distinctive angiogenic features such as increased microvascular blood content, irregularity of the microvasculature and increased microvascular volume [55–57]. The progressive architectural distortion with tumour neo-vasculature consists of precocious capillary sprouting, convoluted and extensive vessel branches, and discontinuous and wider endothelial junctions between cells. These abnormalities result in irregular flow patterns, higher intravascular pressures, and extravascular leakage [58–61]. Sensitive determination of tissue microvasculature using ICG can visually discriminate these differences dynamically and so differentiate invasive from non-invasive neoplasia [62] (and more generally healthy tissue from non-healthy tissue) as each tissue type exhibits a specific vascular pattern based on its architecture (see Fig. 1). Empirical evidence for this has been seen by several groups [63–65]. We ourselves have previously reported on in-vivo assessment of primary colorectal cancers following intravenous administration of ICG. When observed continuously in situ for up to ten minutes, the rate of inflow in cancerous tissue is slower relative to adjacent normal tissue [66]. Active and passive nonspecific processes as detailed above likely play additional roles in tumour *retention* after initial perfusion [42, 47–49] and result in differential and in fact discriminant inflow/outflow behaviours. While such behaviours may be observable to the naked eye, ICG flow through benign tissue may appear similar making it difficult and burdensome to make an accurate prediction from visual assessment alone. Such behaviours therefore are better captured by quantitative time series extraction as illustrated in Fig. 2. The differences in the curves produced from ICG perfusion analysis of rectal cancer may be subtle. Hence, their analysis and tissue status prediction is best carried out by machine learning classification algorithms. This differs to other examples of dynamic perfusion analysis where differences are clearer to the naked eye [67–69]. The same underlying temporospatial patterns related to ICG flux are observable in microscopic sections taken at different time points after ICG administration when examined by near infrared microscopy alongside standard pathological staining (see Fig. 3, and note these images also illustrate how the physiological processes involved are not entirely explainable by EPR alone).

### Dynamic perfusion-based imaging in radiology

Characterisation of lesions in-situ by their perfusion patterns is an established concept in radiology that has been exploited in dynamic contrast imaging for decades, particularly Magnetic Resonance Imaging (MRI) and Computed Tomography (CT). Dynamic contrast enhanced MRI (DCE-MRI) is utilised across a wide variety of organs including breast, prostate, and liver with high levels of accuracy for cancer detection. Particularly in liver malignancies, DCE-MRI accuracy has reached levels that often preclude the need for biopsy [70, 71]. Focussing on the transfer constant (K^Trans^) in DCE-MRI for rectal cancers, we can see that it correlates with angiogenesis, vascular endothelial growth factor levels, tumour differentiation, tumour aggressiveness in rectal cancers and may predict response to neoadjuvant therapies [72, 73]. Recently, *Muto et al.* reported that K^Trans^ levels have also shown a correlation with increased ICG retention in pituitary tumours and intracranial meningiomas [74, 75]. The cell level factors which dictate K^Trans^ levels in DCE-MRI are the same factors responsible for ICG pooling in rectal cancers, namely permeability and angiogenesis. We have shown that intraoperative tissue characterisation is possible based on interpretable biophysical parameters found in association with these factors [76]. However, there are important differences between the curves produced by DCE-MRI and ICG fluorescence angiography. *Intes et al.*, demonstrated a delayed inflow and outflow of ICG through breast malignancy in comparison to nearby healthy tissue in a similar fashion to that which we have observed in rectal cancers [77]. Such findings, however, are in contrast to those seen in DCE-MRI for breast and prostate cancer where, malignancies are generally seen to peak earlier and wash out more quickly than nearby healthy tissue (Type 3 curves) [68, 69]. This rapid wash-out phenomenon seen in DCE-MRI has been proposed as being secondary to arteriovenous anastomoses causing rapid outflow and therefore a reduction in contrast signal [78], although to our knowledge this has not been proven.

### Intraoperative artificial intelligence (AI) decision support using ICG perfusion characterisation

The biophysical model detailed above is focussed on temporospatial fluorescent patterns and forms the basis of our methods. With this we have begun developing a computational analysis method as a means of real time tissue characterisation potentially obviating forceps biopsy and enabling margin delineation. In comparison to standard biopsies and histopathological analysis, what is exciting about ICG fluorescence signals is the production of an in-surgery assessment of the entire tumour (including some millimetres below the mucosal surface). Initially such an ICG perfusion-based decision support method could be used to direct tissue biopsy to the area of abnormality most likely to contain any cancerous component and thereafter, should AI based digital assessment outperform endoscopic biopsy in ongoing validation studies [79, 80], the need for tissue sampling could be avoided. Even just equalling the accuracy of biopsy would be a significant advancement, providing a similarly accurate prediction to patients on the day of their investigation. In a similar manner, an assessment of overall tumour size and on-screen margin guidance would be greatly beneficial for endoscopic mucosal, submucosal and full thickness local resections, encouraging optimised dissection at the index procedure and reducing involved margin status [81–83].

So far, our methods have produced accuracy results in the region of 90% for cancer discrimination in significant (i.e. those over 2 cm in diameter) rectal polyps and small cancers [76, 84]. To do this, ICG curve features based around parameters reflective of the wash in and wash-out phases of ICG need to be extracted from intraoperative surgical imaging both from the area of observed abnormality identified (for instance a rectal polyp) and an adjacent area of normal tissue viewed with the same field of view simultaneously. Such features have previously been applied to perfusion (but not cancer) quantification in animal models [85]. The resulting feature profiles then form the foundation for AI classification perceptron prototypes after appropriate weighting and application as a formula to enable specific prediction of a ground truth being in this instance whether the area of interest contains cancer or not in the final pathology report after lesion excision (for an example of one such formula see reference [76]).

**Fig. 4:**
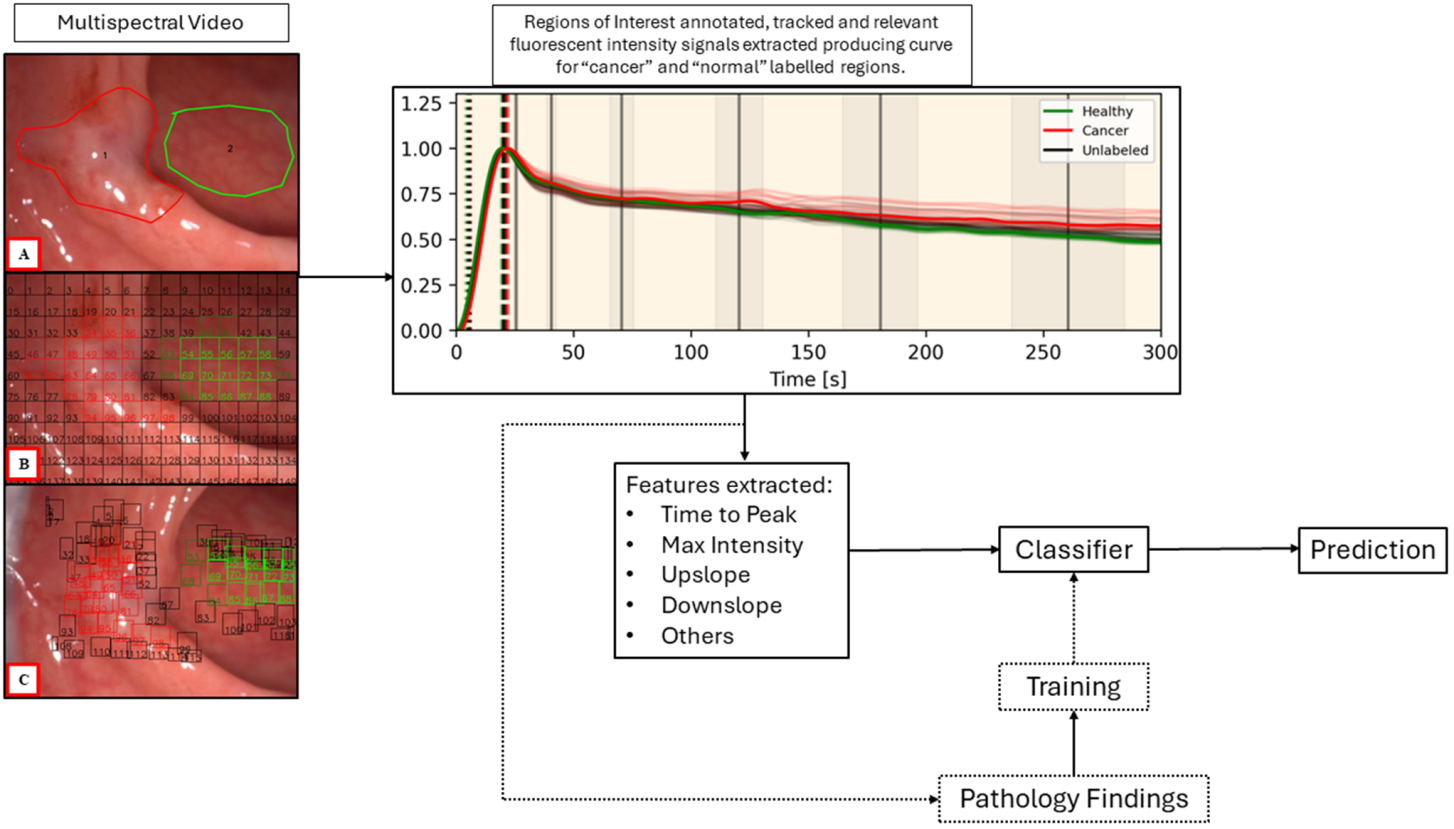
Schematic showing the end-to-end AI computing method proposed following endoscopic lesion classification by a surgeon or gastroenterologist. Image **A** shows the annotated areas with the suspicious lesion within the red border and healthy control outlined by green. Images **B** and **C** show the first and last frames respectively with tracked regions. With image stabilisation, analytical grids are applied to the image and analysed for ICG inflow by evidence of their fluorescence intensity, producing corresponding curves. Trained mathematical models are deployed to turn the curve features into a formula that outputs a prediction regarding cancer likelihood in comparison to that of an area without disease visible on the same screen at the same time

Figure 4 illustrates the end-to-end computing developmental method. The lesion being studied is examined with a NIR imaging laparoscopic camera held steadily by the surgeon. Recording begins before the dose of ICG is administered intravenously, ensuring that the time of inflow is detected, and the true upslope can be recorded. Once the video is obtained, the video is annotated by the surgeon with red indicating the area suspicious for cancer and green the normal control. The video is split into 150 regions of interest all of which are tracked at 1 frame per second for 5 min (up to 45,000 data points are extracted per video). Once data is extracted, noise and artefact removed, and curve smoothing completed, a “lesion” vs “normal” graph is produced, and curve features extracted. Curve features are then inputted into the bespoke classifier pipeline which produces a prediction. This pipeline is comprised of multiple, individual, parallel machine learning classifiers (i.e. K-nearest neighbour, support vector model, naïve bayes, etc.) with a voting system aggregating the results of each one. The individual classifiers within this pipeline have also been used for DCE-MRI assessment [86, 87].

To meet the timeframes needed for an intraoperative decision, such prediction needs to happen within seconds or minutes with elevated levels of accuracy and trust. Reliable predictions can now be obtained with approximately 150 s of ICG perfusion monitoring and further shortening is expected by the incorporation of advanced computing power including graphic processing units. The prediction generated may prompt biopsy of a lesion, local resection, or full staging and stratification towards neoadjuvant treatments and/or radical resection. Aside from more rapid processing, there is significant room for improvement within this stratification process. Noise and artefact within the raw curve outputs are significant issues due to patient and surgeon movement and their removal is essential to ensure curve quality. Curve smoothing methods can then be employed (coefficients from the smoothing process may also be utilised in classification). Adequate image stabilisation and tracking are other important prerequisites. Curve features which form the basis for region of interest assessment include TTP, time to half-peak intensity, upslope (rate at which peak intensity is reached), downslope at X seconds (average downslopes between peak and X seconds further), skew and kurtosis (for detailed descriptions of the mathematical methods for accurately calculating these features see Ref [79]). We have also previously reported on our experience with the training of classification models with Leave-One-Out Cross-Validation in smaller datasets followed by five-fold cross validation in larger cohorts. These have shown excellent levels of accuracy (> 86%), outperforming endoscopic biopsy (the current gold-standard) consistently [76, 79, 84]. Our most recent work involves a 100-patient cohort with overall similar levels of performance. This method is dependent on user annotated regions of interest compared to normal control. During its development we also developed full field of view automated classification without user input. This method can provide the surgeon with an in-real-time heatmap overlaid on the intraoperative surgical imagery, with the heatmap indicating regions most likely containing cancerous tissue. This method, trained on earlier region of interest datasets, once again outperforms endoscopic biopsy for the cohort examined (manuscript reporting these methods is currently under submission for publication) and has the benefit of obviating the need for lesion annotation. This development presents an opportunity to look beyond the detection of cancer only, and to explore boundary delineation, quantification of cancer volume within lesions, detection of specific cancer features and alternative/adjunctive methods of classification. An alternative method may lie in an increased variance of fluorescence intensity and time parameters within cancerous and benign lesions in comparison to normal tissue. Given the distorted nature of cancerous vasculature and architecture, we hypothesise that fluorescence signals will be more varied within such lesions [53, 57, 61]. This theory is particularly representative of cancerous polyps, where inflowing ICG will pass from normal to benign tissue before reaching a malignant centre (again, see Fig. 1). Our early analysis has demonstrated a significantly increased variance within cancerous and benign lesions which may serve as a basis for classification. Intratumoural analysis may obviate the need for comparison to a normal tissue control. This may prove useful for polyps in awkward positions and in cases of malignancy developing in the context of inflammatory bowel disease lacking adjacent normal tissue.

This computational method is fundamentally different to other AI methods currently employed during colonoscopy for polyp detection. Such detection systems utilise deep learning detection algorithms such as You Only Look Once (YOLO) detection and region based convoluted neural networks (R-CNN). This contrasts to the simpler machine learning classification methods we utilise [88, 89], which have specific advantages as discussed below. Similar computational methods as our cancer classification method are now also in development for grounded bowel perfusion assessment. An AI-driven intraoperative tool for this application would help surmount learning curve issues and the cognitive burden associated with the subjective evaluation of qualitative dynamic imaging [90].

### Important considerations for fluorescence based cancer characterisation

Access to reliable datasets within medical specialities can be challenging [91]. Biophysics-inspired AI algorithms, such as those that we are developing, need training and testing against a library of clinical surgical videos. However, the number of videos needed is much smaller in comparison to the numbers required for deep learning classification methods seen in radiomics [79]. Such deep learning models often utilise datasets in the region of thousands [92], tens of thousands [93] or even hundreds of thousands [94]. In the context of rectal neoplasia this is especially pertinent, where datasets of these sizes containing video recordings are not available and difficult to compile. A biophysical inspired approach forgoes the need for a vast quantity of videos. Instead, assessment based on fluorescent angiography can yield accurate results with a moderately sized dataset of around 100 cases. While deep learning classification of colorectal polyps based on colonoscopy videos is possible, this method is based on surface appearance only [95]. It is also important to note that rectal cancer most often develops within a pre-existing benign area of neoplasia, so the surface may retain the appearance of a benign lesion of comparable size. This may remain the case even after a considerable proportion has undergone malignant transformation [96]. NIR ICG evaluation penetrates several millimetres into tissue providing beyond-the-mucosa assessment, differing greatly to other light-based endoscopic assessment methods [97]. Potentially, once validated, the fluorescence-based polyp classification may be employed in a synergistic manner with surface features based deep learning methods.

As computer vision and AI methods advance exponentially, bringing with them increased accuracy and possibilities, the medical field risks moving further from explainable decision making. While our methods exploit such advancements, the end-to-end process outputs remain explainable and interpretable. It is important to note the distinction between “interpretable” and “explainable” AI. In interpretable AI it is clear how the inputs provide the outputs (white box). Explainable AI on the other hand can be very different, despite both being often grouped together. Explainable systems are those which utilise a non-interpretable (or black box) system making predictions with a superimposed explanatory algorithm. While the explanation may be sophisticated, there is no way to be sure that it is correct and to truly interpret how the machine made its prediction [98]. Interpretability and explainability, while not absolute requirements for approval by the FDA or other regulatory bodies, are important considerations. The ITFoC (Information Technology for the Future of Cancer) Consortium defines explainability as a key step in their framework for AI validation in precision medicine [91]. Explainability is particularly important for intraoperative assessments where the machine learning is employed with a view to influencing decision making in real time. This contrasts greatly with instances where deep learning is used for detection of potential lesions for endoscopists to then evaluate [89] or where computer aided diagnosis is employed [99], both examples where the outcome may be double checked by the clinician.

### Multicentre validation and generalisability

Validation of the ICG cancer classifier method described here is currently ongoing under a Horizon Europe award (CLASSICA project, NCT05793554) involving other leading surgical cancer centres in Europe [80, 100]. The CLASSICA Project will be carried out in a phased manner with the first phase validating the generalisability of the underlying mathematical concept and building bespoke clinical grade software and optimising the classification system. Subsequent studies will test and broaden the potential clinical applications of this method in real world settings including guided biopsy and marginal guidance for TAMIS excision. Beyond purely the clinical validation of the methodology, the acceptability of this method for clinical classification to all stakeholders (including patients) will be explored through surveys, focus groups and reference to existing and proposed legal frameworks. Through collaboration with the European Association of Endoscopic Surgery and the IRCAD training institute in Strasbourg the CLASSICA group aims to position this study as a standard bearer for the use of AI-assisted surgical decision-making tools, informing future clinical guidelines regarding their implementation. With the exponential increase in AI based healthcare tools in recent years there has also been an increase in proposed frameworks for validation [91, 101, 102].

### Beyond cancer/not cancer labelling

Further subclassification of cancers beyond yes/no may be possible from a digital approach, with some early work already correlating biomarker presence with ICG fluorescence. Delta like canonical notch ligand 4 (Dll4) affects angiogenesis within breast cancer and is a potential therapy target. ICG based NIR imaging has been shown to accurately predict DII4 levels with a sensitivity and specificity of > 90%, in a non-invasive manner via machine learning [64]. This concept has potential for stratifying patients for targeted therapies. There is also a potential use for such an AI-ICG method in prediction of antibody pharmacokinetics in solid tumours. Pharmacokinetic model analysis has shown that monoclonal antibody (mAb) tumour disposition is influenced by passive transport processes such as capillary permeability [103, 104]. These passive transport processes cannot be assessed on histopathological biopsies [105]. Instead, DCE-MRI or ICG with NIR are viable options to assess vascular permeability and passive transport. Bordeau et al. showed recently that the addition of K^trans^ to physiologically based pharmacokinetic modelling improved the accuracy of mAb tumour disposition in a mouse model [105]. At a minimum we hypothesise that assessment of the ICG signal from individual rectal tumours would provide insight into T stage as well as indicate tumour differentiation, vascularity, and stroma component which in turn are measures of cancer subtype in a disease that currently lacks an in vivo molecular characteriser.

### Optical imaging system considerations

NIR camera systems also make some adaptions to the signals as the images are being produced for the purposes of optimised, consistent display for surgeon interpretation, which need to be considered and catered for during processing [106] including certain factors such as centre-periphery brightness differences. *Leiloglou et al.*, reported in 2021 on their development of a two-camera system for fluorescence guided surgery which allowed for sub-mm microvasculature assessment via ICG angiography [40]. This study also capitalised on fluorescence image texture metrics, as proposed by *Streeter et al.* [107], further improving diagnostic accuracy in breast conserving surgery [40]. Sub-mm angiography would provide a significantly more detailed dynamic assessment of rectal lesions, which is likely beneficial for retrospective analysis of videos. Intuitively, one would think that such high-fidelity images will most likely produce improvements in real time. Potential small gains in accuracy must be balanced against increased processing times and the need for an intraoperative decision. However, if Moore’s law of computing proves to be true, sub-mm assessment may be possible in the near future. As mentioned above, NIR imaging is only currently available in rigid laparoscopes and only rectal lesions are within reach during natural orifice procedures with this equipment. Evaluation in these cases is usually performed under general anaesthesia with the patient in lithotomy position. Research grade NIR equipped flexible endoscopes have been developed [108–110] and may enable expansion of this methodology beyond rectal tumours in reach of a rigid endoscope including into the upper aerodigestive tracts.

### Next generation fluorophores for intraoperative imaging

ICG could soon be joined in clinical care by additional “selective” near infrared cancer tracers [111, 112]. Colorectal cancer specific fluorophores include SGM-101 targeting carcinoembryonic antigen (CEA) [113] and bevacizumab-800CW targeting vascular endothelial growth factor alpha (VEGF-A) [114, 115] amongst others [111, 112]. Such selective fluorophores offer a potentially higher tumour-to-background ratio (TBR i.e. the ratio of mean tumour fluorescence intensity to that of normal tissue, with a TBR > 1.5 preferable for optimal tumour identification) [111]. Therefore, specific fluorophores could be promising for the identification of tumour margins and small and otherwise occult (e.g. peritoneal) deposits in advanced cancers. SGM-101 has displayed a TBR of 1.9 intraoperatively, while also helping identify malignancies not visible with white light only and identifying benign tissue as benign when it initially appeared suspicious for malignancies [8]. Both agents have led to changes in patients’ peritoneal cancer index (PCI) in phase 2 studies, however they were also associated with high false-positive rates [115, 116]. While these, and most new agents in development are being advanced in the paradigm of antecedent dosing, intraoperative identification will still be required. This is likely to be helped by computer assisted methods of visualisation, especially as many of the agents in development demonstrate some non-selective binding in vivo. The observations and computing methods presented above are directly applicable to other intended intraoperative imaging agents as, at their core, they are all discriminating contrast against background to disclose information about the underlying tissue status. Different imaging strategies may need different AI assistance to maximise as those with weaker signals may benefit from some signal boosting while dynamic assessment augmentation is more likely beneficial for early look assessment following administration versus later examination when the dye is in relatively stable states. Ongoing evolution of ICG imaging will help broaden the clinical user community and encourage further interest in investment in fluorescence guided imaging globally.

## Conclusion

The advent of AI presents an opportunity to further advance the clinical use of ICG and fluorescence guided surgery, as well as develop understandable decision support tools for surgery built on the described method for automatic endoscopic differentiation. The CLASSICA Project, which is currently enrolling patients across Europe, aims to validate these techniques with more to be explored in the form of detailed tissue characterisation, predicting response to neoadjuvant therapy, stratifying patients to personalised therapies, and established decision support tools to improve the performance of surgeons and endoscopists [80]. While this review and our current body of work focusses mostly on rectal cancer, we expect that these principles are transferrable to malignancies throughout in the colon, GI tract and elsewhere. The foundational concepts of these methods are likely applicable to intraoperative cancer characterisation in general. Different tissues, dyes and diseases may demonstrate differences in the inflow/outflow specifics and so different models will need to be trained and tested using different datasets [18]. There is also of course exciting potential for synergy with radiological methods for both diagnosis and therapeutic image guided interventions in the current era of increasing organ preservation via multimodal oncological approaches. With new fluorophores emerging from clinical studies, the importance of highly discriminant intraoperative realisation and action will encourage further synergy with computer vision and AI methods. Such developments may supplement and, in time perhaps, provide some component automation, or basis for replacement, of current surgical therapy for malignant disease which is predicated on removal of the lesion for the provision of pathological characterisation as well as local cure.

## Data Availability

Data sharing is not applicable to this article as no datasets were generated or analysed during the current study.
